# Chemotactic self-caging in active emulsions

**DOI:** 10.1073/pnas.2122269119

**Published:** 2022-06-09

**Authors:** Babak Vajdi Hokmabad, Jaime Agudo-Canalejo, Suropriya Saha, Ramin Golestanian, Corinna C. Maass

**Affiliations:** ^a^Department of Complex Fluids, Max Planck Institute for Dynamics and Self-Organization, 37077 Göttingen, Germany;; ^b^Institute for the Dynamics of Complex Systems, Georg August Universität, 37077 Göttingen, Germany;; ^c^Department of Living Matter, Max Planck Institute for Dynamics and Self-Organization, 37077 Göttingen, Germany;; ^d^Rudolf Peierls Centre for Theoretical Physics, University of Oxford, Oxford OX1 3PU, United Kingdom;; ^e^Physics of Fluids Group, Max Planck Center for Complex Fluid Dynamics and J. M. Burgers Center for Fluid Dynamics, University of Twente, 7500AE Enschede, The Netherlands

**Keywords:** chemotaxis, active matter, caging, microswimmers, self-propelling droplets

## Abstract

A common feature of biological self-organization is how active agents communicate with each other or their environment via chemical signaling. Such communications, mediated by self-generated chemical gradients, have consequences for both individual motility strategies and collective migration patterns. Here, in a purely physicochemical system, we use self-propelling droplets as a model for chemically active particles that modify their environment by leaving chemical footprints, which act as chemorepulsive signals to other droplets. We analyze this communication mechanism quantitatively both on the scale of individual agent–trail collisions as well as on the collective scale where droplets actively remodel their environment while adapting their dynamics to that evolving chemical landscape. We show in experiment and simulation how these interactions cause a transient dynamical arrest in active emulsions where swimmers are caged between each other’s trails of secreted chemicals. Our findings provide insight into the collective dynamics of chemically active particles and yield principles for predicting how negative autochemotaxis shapes their navigation strategy.

## Significance

The out-of-equilibrium dynamics of chemotactic active matter—be it animate or inanimate—is closely coupled to the environment, a chemical landscape shaped by secretions from the motile agents, fuel uptake, or autochemotactic signaling. This gives rise to complex collective effects, which can be exploited by the agents for colony migration strategies or pattern formation. We study such effects using an idealized experimental system: self-propelled microdroplets that communicate via chemorepulsive trails. We present a comprehensive experimental analysis that involves direct probing of the diffusing chemical trails and the trail–droplet interactions and use it to construct a generic theoretical model. We connect these repulsive autochemotactic interactions to the collective dynamics in emulsions, demonstrating a state of dynamical arrest: chemotactic self-caging.

Motile microorganisms have evolved to sense their environment and react to external chemical or physical cues via taxis. Specifically, organisms respond to a gradient in the concentration field of a chemical species by chemotaxis (1) or autochemotaxis when the gradient is generated by the organisms themselves (2). In microorganisms, chemotaxis and autochemotaxis guide many collective processes, such as colony migration (3, 4), aggregation (5, 6), or biofilm formation (7), where the emergent complex behavior is governed by the interplay of physical effects and biological processes. Many aggregatory, quorum-sensing (8) behaviors are based on attractive signaling (i.e., positive autochemotaxis). Repulsive signaling (negative autochemotaxis) is of practical importance to efficient space exploration (e.g., when ant colonies forage using mutual avoidance) (9).

Complex collective behavior can result from intricate biological mechanisms but also, can be solely caused by nonequilibrium dynamics (refs. 10–14 have such examples), such that there is a need to untangle physics and biology. To this end, current research in artificial active matter aims to design and develop synthetic microswimmers that can mimic strategies like chemotaxis by purely physicochemical means (15). Self-phoretic particles, which propel via a self-generated local chemical gradient (16–18), are widely studied in theory and experiment. Suspensions of these particles exhibit nontrivial dynamics influenced by autochemotaxis (19–24). Specifically, self-propelling droplets (25) provide an experimental model for repulsive chemical signaling (12, 26–28). Along their way, the droplets shed a persistent trail of depleted fuel, which acts as a chemorepellent to other droplets. Hence, the motion of such a droplet is affected by the previous passage of another droplet.

In this study, we show that in an active emulsion, droplets remodel their chemical environment while adapting their dynamics to that evolving resource landscape. In spirit, this resembles *Pseudomonas aeruginosa* organizing their interactions by shedding attractive trails (7, 29), with the difference that droplet trails are chemorepulsive. We start by a quantitative analysis of individual “delayed collision” events in quasi–two-dimensional (quasi-2D) confinement; we directly visualize and map the chemical footprints of droplets and measure the diffusion coefficient of the constituent chemicals. We use these results to fit a generic analytical model in 2D based on time delay, angle of impact, and chemical coupling strength. We show that these parameters determine whether a droplet crosses a chemical trail or rebounds from it. We then proceed to the collective dynamics, comparing experimental data with simulations using our single-event fits. We demonstrate how such individual binary collisions cause autochemotactic arrest in ensembles of chemically active droplets, a kind of “history caging,” where swimmers are transiently trapped in an evolving network of repulsive trails. We finally address the question of whether this type of caging is possible in three dimensions (3D) via experiments in density-matched bulk media.

## Results

### Self-Propulsion by Solubilization.

**Fig. 1.**
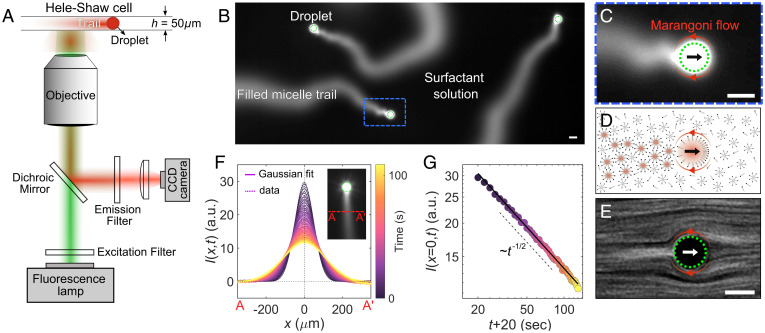
Visualization of the chemical trail. (*A*) Schematic of the experimental setup for fluorescent microscopy of the filled micelle trail. CB15 droplets of diameter $a_{\text{drop}}=50\;\mu \text{m}$ were injected into a quasi-2D microfluidic cell (height = 50 µm) and observed using either bright-field or fluorescent microscopy. (*B*) A fluorescence micrograph of the droplet’s chemical trails, with surfactant concentration increased to 15 wt % to improve trail visibility (increased solubilization rate). (*C*) Zoomed-in view of *B*. (*D*) Schematic propulsion mechanism of the droplet. The black arrow shows the direction of motion. (*E*) The flow field generated by Marangoni flow at the droplet interface visualized by streak lines of 0.5-µm fluorescent tracer colloids (droplet reference frame). (*F*) The time evolution of fluorescent intensity (in arbitrary units [a.u.]) profiles along AA′ (*Inset*) superimposed with Gaussian fits (surfactant concentration 5 wt %). (*G*) Peak intensity vs. time. The zero point in time is shifted by 20 s from the droplet passage time to account for the fact that the droplet is not a point source $I_{0}\delta (x)$ (*Materials and Methods*). Green circles mark the droplet boundary in overexposed areas. (Scale bars: 50 µm.).

We study active emulsions containing microdroplets of CB15 oil in a strongly supramicellar aqueous solution of the surfactant TTAB. The gradual solubilization of the oil into the surfactant micelles causes droplet activity. While the solubilization is radially isotropic below a threshold surfactant concentration, above the threshold this symmetry is broken by a spontaneously emerging front–back gradient in the surfactant coverage of the interface, imposing via the respective interfacial tension gradient (Marangoni effect) an orientation ***n*** or polarity onto the droplet and leading to self-propulsion in the direction of ***n***. This can be readily understood if we assume that an increased fraction of oil-filled vs. empty micelles ($\rho_{\text{fm}}/\rho_{\text{em}}$) in the vicinity depletes the interface of surfactant (25, 30, 31). Therefore, the trail of oil-filled micelles in the wake of a moving droplet locally increases the interfacial tension (Fig. 1*D*), providing positive feedback to the front–back tension gradient that leads to self-supporting motion. This sensitivity of the interface to local changes of $\rho_{\text{fm}}/\rho_{\text{em}}$ also applies to the trails left by other droplets; ***n*** is continuously reoriented toward areas of lower $\rho_{\text{fm}}/\rho_{\text{em}}$ (12, 27).

### Quantification of the Chemical Trail behind the Droplet.

To measure this chemical trail, we added the hydrophobic fluorescent dye Nile Red to the oil phase (32) of a droplet swimming inside a quasi-2D microfluidic cell. The dye molecules comigrate with the oil into the swollen micelles left in the wake of the droplet. We recorded and quantified the distribution of oil-filled micelles via fluorescent microscopy (Fig. 1 and Movie S1).

To model the trail diffusion, we approximate the droplet as a point source moving at speed $V_{0}\approx (26\pm 5)\mu \text{m}\;{\text{s}}^{\text{-}1}$ and emitting a fluorescent chemorepellent at a constant rate. Accordingly, the fluorescence intensity along a fixed line perpendicular to the trail (e.g., AA′ in Fig. 1 *F*, *Inset*) should be Gaussian, with a peak height scaling with $t^{-1/2}$. The measured time-dependent intensity along AA′ in Fig. 1 *F* and *G* shows excellent agreement with the Gaussian model, yielding a diffusion constant for filled micelles $D_{\text{fm}}=52.5\mu {\text{m}}^{2}/\text{s}$ (details are in *SI Appendix*). This is further consistent with the Stokes–Einstein relation $D_{\text{fm}}=k_{B}T/(6\pi \eta r_{\text{fm}})=55.2\;\mu {\text{m}}^{2}/\text{s}$ for $r_{\text{fm}}=3\;\text{nm}$ and literature values (12, 33). We estimate the diffusive timescale of the trail spreading as $\tau_{\text{diff}}=a_{\text{drop}}^{2}/D_{\text{fm}}\approx 45\;\text{s}$, which is considerably longer than the droplet’s advective timescale $a_{\text{drop}}/V_{0}\approx 2\;\text{s}$. Such long-lived chemical gradients influence the propulsion dynamics of other droplets even after hydrodynamic interactions have decayed. Beyond the advective timescale, we can, therefore, model droplet–trail interactions by a “dry” chemically active polar particle (CAPP) approach (34, 35). We note that we approximate all hydrodynamic and chemoadvective details into a generic coupling of a local chemical gradient to a point particle. This can be justified on the basis of previous experimental findings (12, 26), which also permit the application of this model not only for droplets but also, for other swimmers, such as Janus colloids and microorganisms.

### CAPP Model.

The fore–aft flow at the interface is determined by the gradient in the local concentration of the empty micelles. We assume that, in the steady swimming state, the fore–aft asymmetry of the droplet is stably maintained. Thus, when a droplet encounters a trail, it experiences an effective torque governed by a coupling constant Ω and an effective force, with coupling constant *α*.

Denoting the position of droplet *i* by $r_{i}$ and its orientation by $n_{i}$, their Langevin dynamics are as follows (34):

[1] $${\dot{r}}_{i}=V_{0}n_{i}-\alpha \nabla c{|}_{r=r_{i}}+\sqrt{2D_{t}}\;\xi_{t},$$

[2] $${\dot{n}}_{i}=\Omega \;n_{i}\times (n_{i}\times \nabla c{|}_{r=r_{i}})+\sqrt{2D_{r}}\;n_{i}\times \xi_{r},$$

where c(r,t) is the micelle concentration field; $\xi_{t}$ and $\xi_{r}$ represent Gaussian-distributed translational and rotational noise with unit strength, respectively; and *D_t_* and *D_r_* are the corresponding translational and rotational diffusion coefficients of the droplet, respectively. The analysis of the mean squared displacement of the droplets in dilute conditions reveals ballistic trajectories over periods longer than 100 s (see Fig. 4*B*), suggesting that Brownian translational diffusion is negligible and that the rotational diffusion coefficient is also small, with an upper bound of $D_{r}=0.01\;{\text{rad}}^{2}/\text{s}$ corresponding to persistence lengths larger than $V_{0}/D_{r}\approx 2.5\;\text{mm}$. We note that the general theory of CAPPs also allows for an effective force projected along the axis of the particle (34), with a third coupling constant, but we will neglect this possibility here as the data can be explained within the more minimal model with two coupling constants.

To model the micelle concentration field c(r,t), we use a simplified static one-dimensional Gaussian profile perpendicular to the direction of motion when studying individual droplet–trail interactions (*SI Appendix*, section 1.G) and a full description of the dynamic 2D concentration field when modeling the collective behavior (*SI Appendix*, section 1.I).

### Quantitative Analysis of Droplet–Trail Interactions.

**Fig. 2.**
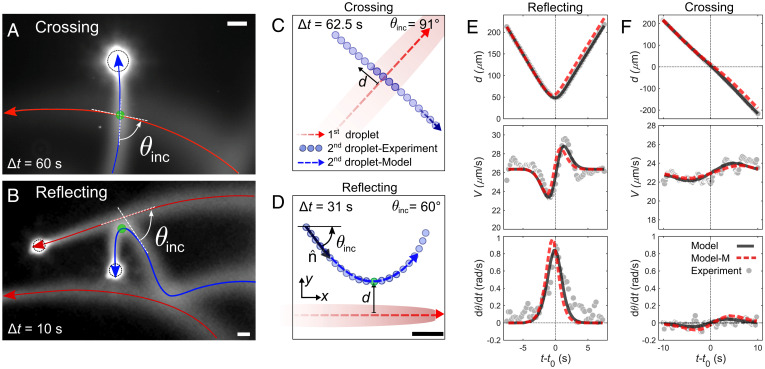
Autochemotactic interaction between a droplet and a trail. Fluorescent micrographs of a crossing (*A*) and a reflecting (*B*) interaction. The red trajectory corresponds to the first passing droplet secreting the trail, and the blue one corresponds to the following droplet. (*C* and *D*) Trajectories from bright-field microscopy for one crossing and one reflecting interaction. Dashed lines are the theoretical fits from the CAPP model using fit parameters $\Omega c_{0}$ and $\alpha c_{0}$. (*E* and *F*) Plots of signed distance *d*, swimming speed *V*, and rotation rate dθ/dt (angular velocity) for typical reflecting (*E*) and crossing (*F*) interactions, respectively. Black lines (model) correspond to the best fit for the given trajectory, and red dashed lines (model M) correspond to the theoretically predicted trajectories using the median values of all fits analyzed, $\Omega c_{0}=7\cdot 10^{3}\;\mu {\text{m}}^{2}\text{/s}$ and $\alpha c_{0}=3\cdot 10^{4}$ $\;\mu {\text{m}}^{3}\text{/s}$. (Scale bars: 50 µm.).

We now proceed to study the individual droplet–trail interactions in a quasi-2D microfluidic geometry (details are in *SI Appendix*, section 1.E). Fig. 2 shows examples of crossing (Fig. 2*A*) and reflecting (Fig. 2*B*) events (Movies S2 and S3). The following droplet (blue trajectory) approaches at an incident angle $\theta_{\text{inc}}$ with respect to the first trajectory (red). An interaction starts and ends when the distance |d| between the droplet and the trail falls below a threshold value $d_{\text{max}}=220\;\mu \text{m}$. We identify the points of intersection (green points) or for reflection, the closest approach on each trajectory, and we define the time lag Δt as the interval between each droplet passing these points and the time origin *t*_0_ as the respective point in time for the following droplet. We observe, for similar $\theta_{\text{inc}}$, a transition from reflection to crossing with increasing Δt.

To match the CAPP model to the experimental data, we analyzed delayed collisions, as shown in the examples in Fig. 2 *C* and *D*. For the following droplet, we measured the signed distance *d* from the trail, speed *V*, and angular velocity |dθ/dt| (Fig. 2 *E* and *F*). We fit the theoretical trajectories to the experimental trajectories in order to estimate the two unknown coupling constants $\Omega c_{0}$ and $\alpha c_{0}$, which were used as fitting parameters (*SI Appendix*, section 1.G has details). Examples of the fits are shown in Fig. 2 *E* and *F* for two experimental trajectories. Here, black lines correspond to the actual fit to the given trajectory, and red lines correspond to the theoretical prediction using the optimal median values obtained from all fits in the dataset: $\Omega c_{0}=7\times 10^{3}\;\mu {\text{m}}^{2}/\text{s}$ and $\alpha c_{0}=3\times 10^{4}\;\mu {\text{m}}^{3}/\text{s}$. The characteristic features of the evolution of the position, velocity, and angular velocity with time are well recapitulated by the model both for each particular fit and when using the median values. Importantly, Ω>0 implies that the droplet reorients to point away from the trail, whereas α>0 implies that the droplet is also directly repelled by the trail. While we found that the trajectory shapes are most sensitive to changes in Ω, which is the key parameter governing the interaction, the presence of a positive *α* is essential in order to correctly capture the time evolution of the droplet velocity (Fig. 2 *E*, *Middle*), which decreases before the turning point and increases after it.

### Incidence Angle and Time Lag Determine the Interaction Dynamics.

**Fig. 3.**
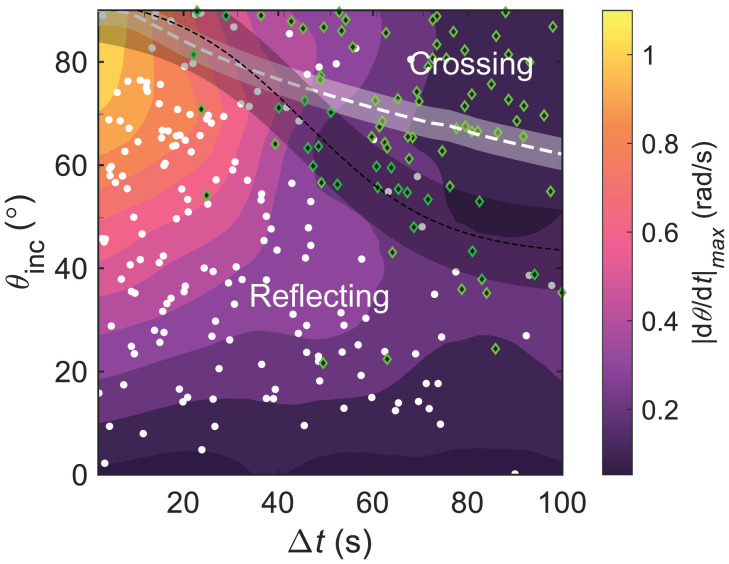
Autochemotactic interaction diagram mapping 164 trail reflections (white) and 90 crossings (green). The interpolated background color corresponds to the maximum rotation rate $|\text{d}\theta /d\text{t}{|}_{\text{max}}$ measured for each interaction. The dashed white line is the separatrix between crossing and reflection as calculated from fits to the CAPP model with a shaded error interval accounting for rotational diffusion; the dashed black line is an estimate of the experimental separatrix between the reflecting and crossing events with an error interval obtained from a sigmoidal fit to the coexistence region (*SI Appendix*, section 1.H).

Investigating the autochemotactic interactions revealed that the probability of crossing vs. reflection depends both on $\theta_{\text{inc}}$ and on Δt, which determine the gradient strength. We extracted $\theta_{\text{inc}}$ and Δt for all 254 identified interactions and plotted them in the interaction diagram in Fig. 3, with reflections marked by white data points and crossings marked by green data points. The background color map interpolates the maximum recorded angular velocity. We can confirm two key features. For one, crossing is more likely for increasing Δt (i.e., decreasing ∇c). Second, for increasing $\theta_{\text{inc}}$ (i.e., sharper reorientations), reflection requires a higher turning rate mediated by Ω|∇c|, such that crossing is more likely for increasing $\theta_{\text{inc}}$. The black dashed line corresponds to the separatrix between the reflecting and crossing events (*SI Appendix*, section 1.H has details).

We now construct a theoretical interaction diagram using the median values of $\Omega c_{0}$ and $\alpha c_{0}$. The separatrix (dashed white line) between crossing and reflecting events, which coincides with those trajectories for which the turning point is exactly at the center of the trail (*y* = 0), reproduces the salient features of the experimental observations. Notably, the model captures the correct order of magnitude of the timescale at which reflection switches to crossing, even if it was calibrated only by fits to individual reflecting trajectories. The small amount of rotational noise present in the system can cause some uncertainty in the location of the separatrix, which we estimate from the SD of the orientation while the incoming droplet approaches the trail: $\delta \theta \approx \sqrt{2D_{r}T}$ using an approach time $T\approx d_{\text{max}}/(V_{0}\sin\theta_{\text{inc}})$ and an upper bound of $D_{r}\approx 0.01$ rad^2^/s for the rotational diffusion coefficient. The strongest deviations between the theoretical prediction and experimental results are observed at low $\theta_{\text{inc}}$ and large Δt>50, in which case both crossing and reflecting trajectories are seen to coexist experimentally. In this region, the incidence angle is shallow, and the required torque is weak. Thus, higher-order effects become significant (e.g., the fact that the trajectory width increases in the wake of the leading droplet [which breaks the symmetry between parallel and antiparallel trajectories] or the remaining trail curvature). Moreover, there is additional experimental variation from the full chemical history of the sample, the small polydispersity of the droplets, and the nonzero rotational diffusivity. However, these cases with large Δt become infrequent with increasing number densities, such that a quantitative mismatch between experiment and theory for large Δt would have no strong bearing on the caging effects modeled in the following section.

### Collective Dynamics Governed by Autochemotactic Interactions: History Caging.

**Fig. 4.**
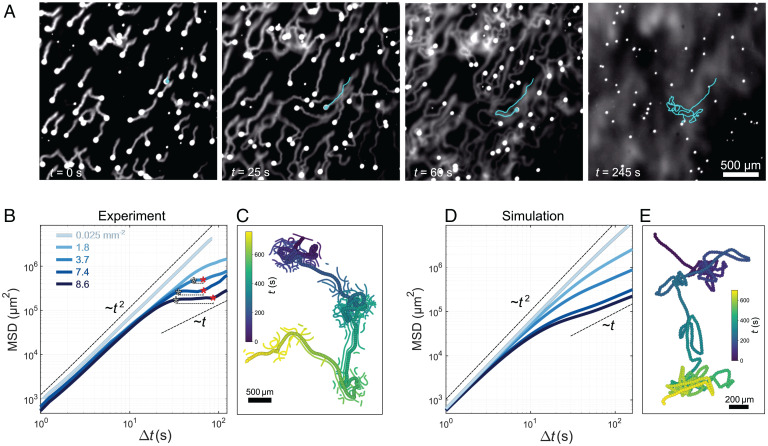
Caging in 2D. (*A*) Snapshots from fluorescent microscopy of an active emulsion in a quasi-2D cell (droplet size 50 μm) under high droplet solubilization rate to increase trail fluorescence. The cyan trajectories demonstrate the evolution from ballistic propulsion to caging. (*B*) Mean squared displacements for emulsions with increasing number densities. The black asterisks denote the cross-over to the caging regime, and the red asterisks denote the cross-over to the cage-escape regime. (*C*) A trajectory *s*(*t*) with consecutive caging and cage-escape events (thick symbols). Thin symbols indicate other droplets detected within a spatiotemporal window of d<220 µm and $0<t_{0}-t<50\;\text{s}$ around the current trajectory point $s(t_{0})$. Data are color coded by time. (*D*) MSD and (*E*) an example trajectory obtained from simulations of the CAPP model under the same conditions as the experiments in *B* and *C*, demonstrating similar caging behavior.

To study the consequences of autochemotactic interactions in a crowded system, we placed suspensions of swimmers at number densities *n* between 0.025 and 8.6 mm^−2^ in a quasi-2D cell and recorded their trajectories for longer times (≈5 min). We first illustrate the collective behavior using a fluorescently dyed sample in Fig. 4*A* (Movie S4) at increased solubility for better visualization. Initially, all droplets move persistently but reorient when they encounter a trail. Gradually, the secreted trails form a network. A complex chemical landscape, based on ∇c, evolves in time and space with multiple local minima (dark regions) between the trails. The swimmers are trapped in these interstitial minima by multiple reflections at the cage walls (Movie S5), but the caging is transient since droplets can escape either when ∇c has decreased sufficiently at the cage boundaries or when the chemical buildup caused by the droplet itself forces it out of the cage (Movie S6). This kind of arrest by purely chemorepulsive interactions can be described as “chemotactic self-caging” or history caging since the trails constituting the cage walls could be described as the memory of other particles as opposed to classical caging, which is driven by real-time collisions with other particles forming the cages. Remarkably, this type of caging happens at extremely low number densities compared with the conventional caging of glassy systems.

We now quantify the collective dynamics under the same chemical conditions as the droplet–trail interaction experiments. Fig. 4*B* shows, for increasing number densities, the mean squared displacement (MSD) obtained by ensemble averaging over the trajectories:

[3] $$\text{MSD}(t)=\frac{1}{N}\sum_{i=0}^{N}{\left(r_{i}(t)-r_{i}(t_{0})\right)}^{2},$$

where *t*_0_ is the starting time of the experiment and *N* is the number of droplets. For any number density, droplets initially move persistently, with $\text{MSD}∼t^{2}$. For an isolated droplet, we do not see a transition to diffusive scaling ∼t on our experimentally accessible length scales. For intermediate number densities, we observe a change in the slope of the MSD, which is associated with the reorientations caused by the autochemotactic interactions. At large number densities (n≥3.7 mm^−2^), after a short ballistic period the MSD reaches a plateau, which is a signature of caging. Such a plateau is reminiscent of the caging phenomenon in colloidal glasses (36). However, here the caging is caused by trail–droplet interactions instead of direct interparticle collisions and is, therefore, observed at much lower volume fractions ($\phi_{\text{droplets}}\approx 10^{-2}$) (see also ref. 37). For more crowded systems, the cross-over to caging happens earlier, the lifetime of the cage is longer, and the cage size is smaller. The plateau is followed by a cross-over to a third subballistic regime corresponding to the droplet escaping the cage. We demonstrate this for an example trajectory in Fig. 4*C* (thick markers), where a droplet undergoes three caging events (at $n\approx 7.4\;{\text{mm}}^{-2}$). To highlight the cage formation by spatiotemporal aggregation, we plotted all droplet positions (thin symbols) recorded within a window of d<220 μm and 0<Δt<50 s around each current droplet position. Entering an area with increased density, the droplet reorients frequently, exploring the cage, until it is ejected due to the gradual buildup of chemorepellent into a less populated space (“cage escape”), where it proceeds persistently until it encounters the next high-density area (see also Movie S6).

The largest cage size that we observe in Fig. 4*B* is $l_{\text{cage}}\approx 500\;\mu \text{m}$. Given the average swimming speed of $V_{0}\approx 25\;\mu \text{m/s}$, we can estimate the longest Δt during caging experiments to be $l_{\text{cage}}/V_{0}\approx 20\;\text{s}$, which falls well within the region of good agreement between experiments and theory in Fig. 3. We recover the caging behavior in numerical simulations of the CAPP model (*SI Appendix*, section 1.I has details) using only the experimentally measured parameters as well as the median $\Omega c_{0}$ and $\alpha c_{0}$ extracted from the fits to individual particle–trail interactions without any adjustable parameters (Fig. 4 *D* and *E*). Based on the linear coupling between chemorepellent gradient and forces/torques (12), we can assume the effects of individual trails to simply superimpose. We find a subdiffusive interval at the same particle densities and with comparable timescale (∼20 to 80 s) and length scale ($∼10^{5}\;\mu {\text{m}}^{2}$) as for the caging observed in experiments. Beyond the caging regime, the MSD transitions to a diffusive scaling as expected.

### Caging in 3D.

An intriguing question is how far dimensionality matters in the emergence of caging. In 2D confinement, without trail memory loss, fully confining cages exist even for one-dimensional trails with vanishing width. In 3D, such a cage would always have holes the droplet can escape through. However, the superposition of multiple trails diffusing in 3D space (*SI Appendix*, Fig. S4) would still create a highly inhomogeneous field of chemorepellent, where droplets can be trapped in local minima.

**Fig. 5.**
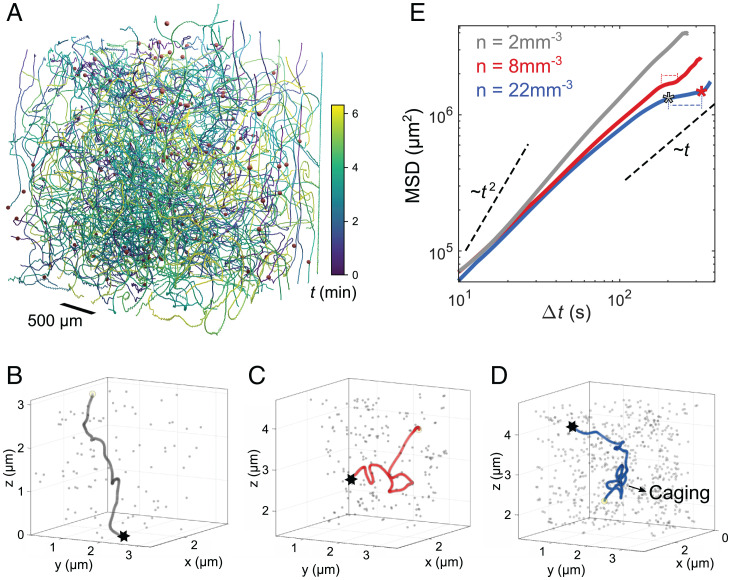
Caging in 3D. (*A*) Tracking by fluorescent light sheet microscopy; 3D reconstruction of trajectories recorded over ≈6.3 min and the droplet arrangement (red spheres) after 29 s at number densities $n=8\;{\text{mm}}^{-3}$. Trajectories are color coded by time. Sample volume (3 mm)^3^, swimming medium, and droplet density are matched by ${\text{D}}_{2}\text{O}$ admixture to enable force-free swimming. (*B–D*) 3D trajectories for one droplet swimming in emulsions with increasing number density 2, 8, and 22 mm^−3^ recorded during 221, 380, and 490 s, respectively. End points are marked by ⋆. Gray markers show one typical arrangement of all droplets. (*E*) Mean squared displacements of 3D active emulsions with increasing *n*. Asterisks mark crossovers as in Fig. 4*B*.

To investigate this mechanism of caging in 3D, we placed active emulsions of varying number densities *n* in a swimming medium density matched by heavy water admixture. We recorded the 3D trajectories of droplets inside a (3-mm)^3^ volume using a scanning light sheet fluorescence microscope, as shown in Fig. 5*A* by 3D rendering of trajectories recorded over ≈6.3 min and the droplet arrangement after 29 s at $n=8\;{\text{mm}}^{-3}$. We plot typical trajectories for systems with increasing *n* in Fig. 5 *B*–*D*. In the dilute case, $n=2\;{\text{mm}}^{-3}$, the trajectory is quite straight, while at intermediate $n=2\;\;{\text{mm}}^{-3}$, the droplet undergoes a few reorientation events. The trajectory for the densest system at $n=22\;\;{\text{mm}}^{-3}$ shows alternating straight and caged sections similar to the behavior in 2D (Fig. 4*D*).

In Fig. 5*E*, we plotted the mean squared displacement of a set of 3D trajectories extracted from the datasets used for Fig. 5 *B*–*D*. The signatures of caging can be observed in the form of plateaus in the MSD, in particular for $n=22\;\;{\text{mm}}^{-3}$.

## Discussion

We have used active emulsions to explore the collective behavior of active particles governed by negative autochemotaxis. Using fluorescent imaging in quasi-2D confinement, we visualized and measured the diffusion coefficient of the chemorepellent trails left in the wake of the droplets, a quantity necessary for theoretical modeling of the interactions. We quantified these interactions and established a generic theoretical CAPP model that can recreate the trajectories and predict the outcome of a droplet–trail collision based on the time lag between droplet passages and the angle of incidence. We would like to highlight the fact that the emergent behavior observed experimentally at the collective level is recovered in the theoretical model without the need for any fitting, as discussed above. This suggests that the basic ingredients of the model—and in particular, the alignment interaction (torque) term with a strong value for its coupling Ω—are presenting a faithful minimal theoretical description of the system.

We note that this universal dry active matter description sets limits to the model’s applicability wherever the specific details of the propulsion process become relevant. Droplets are propelled by self-generated hydrodynamic advection–diffusion instabilities in the interface, and changes in the local chemical environment might cause higher hydrodynamic modes that affect the droplet motion in a nonlinear manner (32, 38–42). However, these higher modes are associated with critical Péclet numbers of surfactant transport. Our experiments were conducted at a surfactant concentration barely exceeding the critical Pe of the lowest dipolar interfacial mode, which is equivalent to steady, quasiballistic self-propulsion. We note that we have not observed a significant distortion of the trail of the preceding droplet by a collision event, which further suggests that chemoadvective effects are of second order.

Hydrodynamic feedback would need to be considered for Δt below the advective timescale, but this type of collision is very rare at the typical number densities relevant in trail-mediated caging and would be more relevant for conventional active glasses (43). While it is in principle feasible to treat individual droplet–trail interactions including hydrodynamic feedback (44, 45), our results show that the CAPP model captures essential features of the delayed collisions in dilute systems, where typically, the time lag between droplet passages is considerably longer than the advective timescale. As shown here, this makes the collective behavior accessible to numerical and analytical modeling using a similar generalized CAPP approach.

On the other hand, owing to the generality of the theoretical framework, the current approach and the insights obtained from this study can be extended to similar systems, where active agents locally deplete the resources and in turn, remodel their environment. The interactions of the agents with the evolving resource landscape (46) result in the emergence of complex collective states, such as adaptive search strategies (47), field-driven localization for ecology-inspired robots (48), or the mechanism for dynamical arrest that we document in this study: repulsive autochemotactic caging in both quasi-2D and unconfined 3D geometries.

Our observations show that, even in 3D, droplets can get trapped in an evolving chemical landscape created by the trails of other swimmers. Their dynamics resemble the recently reported bacterial hopping and trapping in a heterogeneous porous medium (49), however, in this case, the heterogeneous medium is self-generated. Remarkably, we see an enhancement of density fluctuations reminiscent of quorum sensing (50), even based on purely repulsive interactions.

In the description of our experimental findings, the generalized CAPP model has predictive power at the collective level, as it reproduces the timescale and length scale of the experimental caging behavior as well as the critical droplet density at which caging sets in. Compared with 2D confinement, 3D caging sets in at significantly lower volume fractions ($\phi \approx 10^{-4}$). We hypothesize that in this case, the droplet motion in 3D is not rectified by the cell boundaries and that reflection is, therefore, effected by weaker gradients. An in-depth answer to this question will require further quantitative modeling of the individual interactions in 3D.

To put our observations into the context of current research, we note that localization has been found in biological collectives with attractive trails [e.g., in the formation of microcolonies by *P. aeruginosa* (29) or in foraging ants communicating by pheromones (51) as well as in generalized simulations (52)]. It would be of interest to future study to investigate phoretic swimmers with attractive interactions, but at the current state of the art, chemoattraction seems to be incompatible with individual self-propulsion (31, 53).

We further found it noteworthy in our experimental observations that the same chemorepulsive mechanism that causes an enhanced search behavior (i.e., self-avoiding walks) in individual swimmers with increased rotational noise (32) leads to strong localization in collectives. Previous theoretical investigations of such dynamics focused on an individual lattice-based random walker with impaired memory (54, 55) or a single chemorepulsive droplet (56) and reported both enhanced mobility and transient trapping.

Our study poses questions for future research, such as autochemotactic interactions in 3D; the detailed mechanism of and the criteria for caging in 3D; developing analytical solutions for the collective dynamics, for instance, using mean field theory; exploring the limits of the system in terms of number density and the dilute assumption; and quantifying the autochemotactic interaction in suspensions of active droplets with tunable interactions.

## Materials and Methods

A more detailed description of the materials and methods as well as the theory model and simulation are in *SI Appendix*.

### Chemicals.

We produced stock solutions of active oil-in-water emulsions of CB-15 oil [(S)-4-Cyano-4′-(2-methylbutyl)biphenyl, Synthon Chemicals] in TTAB (tetradecyltrimethylammonium bromide, Sigma Aldrich) surfactant solution in microfluidic flow–focusing devices (PDMS; Dow Corning) at a TTAB concentration of 0.1 wt%. For samples used in fluorescence and light sheet microscopy, we added Nile Red (Thermo Fisher Scientific) to the oil phase.

### Fabrication of Microfluidic Cells for Droplet Production and Observation.

We fabricated microfluidic devices using standard soft lithography procedures, used them to produce monodispersed emulsions of CB15, and stored them in aqueous TTAB solution with a submicellar concentration. We fabricated quasi-2D experimental reservoirs of dimension $13\times 8\times 0.05\;{\text{mm}}^{3}$ via ultraviolet photolithography in SU-8 photoresist (Micro Resist Technologies). A detailed description of the fabrication methods is in ref. 32. We added small amounts of stock emulsion to TTAB solution at the target concentration. We filled reservoirs with this mixture, capped them with glass slides, and observed them under bright-field and fluorescence videomicroscopy. For 3D studies, we filled a $3\times 3\times 3{\text{-}\text{mm}}^{3}$ square glass reservoir with similar mixtures and matched the density of the TTAB solution with that of the oil droplet by adding heavy water. The emulsion was then *z* scanned by a laser sheet and recorded by fluorescence videomicroscopy.

### Data Analysis.

We reconstructed time-dependent 2D and 3D positional data by customized Python routines and quantitatively analyzed the trail fluorescence using custom-written MATLAB codes. All scripts are available on request. We further used Python scripts to mine trajectory data for droplet–trail interactions. Numerical fits of the CAPP model to experimental data for individual collisions were done in MATLAB (*SI Appendix*, section 1.H has details). Using the resulting fit parameters, we simulated the collective behavior by Brownian dynamics (*SI Appendix*, section 1.I).

## Supplementary Material

Supplementary File

Supplementary File

Supplementary File

Supplementary File

Supplementary File

Supplementary File

Supplementary File

## Data Availability

Datasets from videomicroscopy underlying Figs. 3–5 have been deposited in Zenodo (DOI: 10.5281/zenodo.6569553).
