# Supplementary material for: Chemotactic self-caging in active emulsions
Source: Proc Natl Acad Sci U S A. 2022 Jun 9;119(24):e2122269119. doi: 10.1073/pnas.2122269119 (PMC9214524; doi:10.1073/pnas.2122269119)
Supplement: Supplementary File [file pnas.2122269119.sapp.pdf]

Chemotactic self-caging in active emulsions: Supplementary Materials

Babak Vajdi Hokmabad [1], Jaime Agudo-Canalejo [1], Suropriya Saha [1], Ramin Golestanian [1,2,*] and Corinna C. Maass [1,3,†]

[1] *Max Planck Institute for Dynamics and Self-Organization, Am Faßberg 17, 37077 Göttingen and Institute for the Dynamics of Complex Systems, Georg August Universität Göttingen, Germany*
[2] *Rudolf Peierls Centre for Theoretical Physics, University of Oxford, Oxford OX1 3PU, United Kingdom*
[3] *Physics of Fluids Group, Max Planck Center for Complex Fluid Dynamics, MESA+ Institute and J. M. Burgers Center for Fluid Dynamics, University of Twente, PO Box 217,7500AE Enschede, Netherlands*

(Dated: April 22, 2022)

1. MATERIALS AND METHODS

A. Materials and characterization

We study active oil-in-water emulsions, whose oil phase consists of CB-15 (Synthon Chemicals), an isotropic isomer of the common nematogenic oil 5CB. Mono-disperse droplets of diameter $d = (50 \pm 3)$ µm are mass produced in microfluidic flow focusing devices (see [1] for the detailed protocol) with a typical polydispersity of 3-5%. For the purpose of chemical quantification, we dissolved small amounts of the fluorescent dye Nile Red (Thermo Fisher Scientific) in the oil phase. Nile Red does not fluoresce in water, such that we can assume the fluorescent intensity to stem from micelles filled with oil and co-migrating Nile Red. This assumption is supported by the diffusive spread of fluorescence in the trail mapped in Fig. S1 . The swimming medium for all quantitative experiments was a 5wt% aqueous solution of TTAB surfactant (Sigma Aldrich). For force free bulk measurements, the density of 5CB ($\rho_{5\text{CB}} = 1.05\,\text{g}\,\text{cm}^{-3}$) was matched by appropriate heavy water substitution ($\rho_{\text{D2O}} = 1.2\,\text{g}\,\text{cm}^{-3}$).

B. Hydrodynamic and statistical parameters of the active droplets.

We estimate the following parameters of interest:

interfacial tension σ	$\approx 3\,\text{mN/m}$
droplet diameter $2R$	(50 ± 2) µm
typical speed U	(25 ± 2) µm/s
dyn. viscosity μ_{CB15}	138 mPa m
density ρ_{CB15}	$1.03 \times 10^3\,\text{kg/m}^3$
Reynolds number Re	$\approx 10^{-3}$
capillary number Ca	$\approx 10^{-3}$
Péclet number Pe (5 wt% TTAB)	4[1]

Since the Reynolds number for the swimming of the droplet is $\text{Re} = \rho UR/\mu \sim 10^{-3}$, all hydrodynamics are inertialess and Stokesian. Furthermore, the thermal fluctuations ($O(k_\text{B}T/2R_\text{d}) \sim 10^{-16}\,\text{N}$) are negligible in comparison with the hydrodynamic viscous friction ($O(6\pi\mu^o R_d V) \gtrsim 10^{-10}\,\text{N}$).

For $\text{Ca} = \mu U/\sigma \sim 10^{-3}$, the droplet is to high accuracy spherical. The isotropic droplet undergoes spontaneous self-propulsion stemming from an advection-diffusion driven interfacial instability which occurs only if the Péclet number, Pe, is larger than a critical value, $\text{Pe} = 4$. The experimental Pe was calculated using an estimation (Eqn. 1 and Appendix B.2) from [1]. All quantitative data for this study was collected at surfactant concentration $c_{\text{TTAB}} = 5$wt.%, corresponding to $\text{Pe} \approx 4$ (*i.e.* just above the experimentally established onset of self-propulsion) and a temperature of 22 °C. The only exception are the data in Fig. 1B and 1A which were only used for illustration purposes and not calibrated for quantitative evaluation.

C. Methods: observation cell, microscopy and image recording and analysis

Unless noted otherwise, all experiments were done in microfluidic cells using a quasi 2D Hele-Shaw geometry. We fabricated the cells directly from SU-8 photoresist (Micro Resist Technology) on a glass microscope slide of area 75 × 50 mm^2. Rectangular cells with an area of 8 × 13 mm and height of 50μm were filled with a suspension of droplets and sealed with a glass cover slip. No air was entrapped in the cell volume, such that we can assume no-slip conditions at all cell boundaries. To study the droplet-trail interactions and the collective behavior we observed active emulsions under a bright-field microscope (Leica DM4000 B) at a magnification of 2.5x. Videos were recorded at 4 frames per second using a Canon digital camera (EOS 600d) with a digital resolution of 1920 x 1080 px. The droplet coordinates in each frame were extracted by custom Python scripts (available on request) using the libraries numpy, PIL, and openCV. We obtained droplet coordinates via a sequence of background correction, binarization, blob detection by contour analysis, and minimum enclosing circle fits. Droplet trajectories were obtained using a frame-by-frame nearest-neighbor analysis.

To directly visualize the filled micelle trail left in the wake of the droplet, we doped the oil phase NileRed and performed fluorescent microscopy on an Olympus IX73

* ramin.golestanian@ds.mpg.de
† corinna.maass@ds.mpg.de

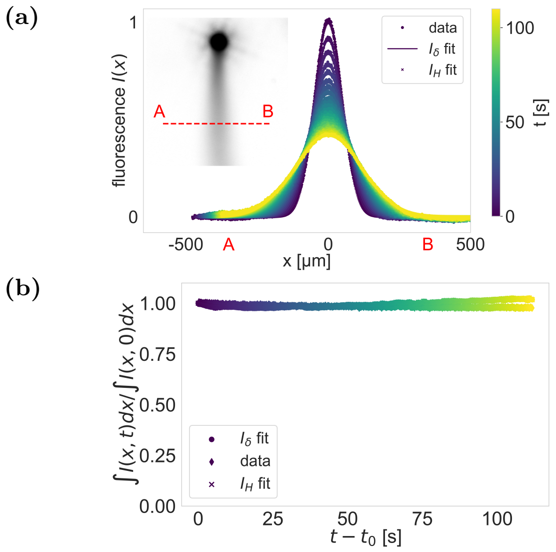

FIG. S1. Fluorescent emission in the trail of a Nile Red doped droplet. (a) Experimental data vs. point source $I_\delta(x,t)$ ((S1)) and stepwise $I_H(x,t)$ ((S4)) emitter fit models. Within experimental precision, the models are indistinguishable. (b) The integrated fluorescence does not decay over time, as shown by numerical integration of the recorded intensity and both fit models.

device with a filter cube (excitation filter ET560/40x, beam splitter 585 LP and emissions filter ET630/75m, all Chroma Technology). Images were recorded using a 4 MP CMOS camera (FLIR Grasshopper 3, GS3-U3-41C6M-C) at 4 frames per second and 4× magnification. Further image analysis to extract intensity profiles and quantify the diffusion coefficient were carried out in MATLAB. We note that, for illustration purposes, the micrographs in Fig. 1B & C, Fig. 4A and Video S4 were recorded at a higher surfactant concentration of 15 wt.% to increase the solubilization rate and thereby the trail fluorescence. However, a lower concentration (5 wt.%) leads to more persistent droplet motion and long cruising ranges and is therefore preferable in quantitative experiments.

D. Micellar diffusion quantification by fluorescence

We recorded fluorescent emission from the diffusing trail as follows. After rotation and rectification of the extracted image series, we extracted profiles along a $y =$ const. image slice (line A-B in Fig. S1 a). We fit the profiles to the time and space dependent decay from a fluorescence quantity $c(x, t = t_0) = M_0\delta(0)$. t_0 is earlier than the actual moment of droplet passage, since the droplet is not a point source:

$$I_\delta(x,t) = \frac{M_0}{\sqrt{4\pi D_{\mathrm{fm}}(t-t_0)}} \exp\left(\frac{-x^2}{4D_{\mathrm{fm}}(t-t_0)}\right), \quad \text{(S1)}$$

$$M_0 = \int_{-\infty}^{\infty} I(x,0)\,\mathrm{d}x \quad \text{(S2)}$$

We calculated the micellar diffusion coefficient via modeling the trail as a linear chemical source that was released and started diffusing t_0 seconds (obtained from fitting) before the passage of the droplet. We then fit the peak values over time to

$$I_\delta(0,t) = \frac{M_0}{\sqrt{4\pi D_{\mathrm{fm}}(t-t_0)}}, \quad \text{(S3)}$$

where $x = 0$ is the center line of the trail.

To cross-check, we also calculated the micellar diffusion coefficient via $\sigma^2 = 4D_{\mathrm{fm}}(t - t_0)$. In principle, a more precise model could be based on a decay from two Heaviside step functions at a distance $d = 50\,\mu\mathrm{m}$, with $c(x, t = 0) = c_0\,[H(x + d/2) - H(x - d/2)]$:

$$I_H(x,t) = \frac{I_0}{2}\left[\mathrm{erf}\left(\frac{x+d/2}{\sqrt{4D_{\mathrm{fm}}t}}\right) - \mathrm{erf}\left(\frac{x-d/2}{\sqrt{4D_{\mathrm{fm}}t}}\right)\right] \quad \text{(S4)}$$

However, beyond the advective time scale the difference between the models is negligible (Fig. S1).

Since the integrated fluorescence does not decay over time in Fig. S1 b, we also do not need to account for possible bleaching effects in our analysis.

E. Binary collisions: Image processing and data analysis

To generate experimental data on droplet-trail collisions for comparison to the CAPP model, we placed droplets at low number densities (0.25 to 0.50 droplets per mm^2) in aqueous surfactant solutions in quasi-2D microfluidic reservoirs. We recorded and analyzed the droplet trajectories via video microscopy and mined the data from 20 experimental runs for suitable trail interactions, as follows. Since our model assumes the preceding droplet to move in a pristine medium with isotropic chemical field, we selected interactions where its motion can locally be well approximated by a straight line (red trajectories in Fig. 2A & B). Thus, we can we assume the chemical gradient in the trail to evolve according to Eqn. S5. After region of interest selection, background correction and binarization, we extracted droplet coordinates via a contour search algorithm [2] combined with a blob size filter for each video frame. Our model does not account for direct hydrodynamic interactions between droplets, for example entrainment. We therefore filtered the resulting coordinate set to exclude droplets whose distance within the same frame are below a set threshold of

$90\,\mu m$, as well as droplets too close to the cell boundaries. Droplet trajectories were extracted from the filtered set via a Crocker-Grier type algorithm [3], providing droplet coordinates x, y and speed vectors $\mathbf{v}$ for each recorded timestep. We identified and analyzed interactions as follows: for each pair of trajectories, we identified matching sections where the trajectory distance fell below a threshold of $220\,\mu m$. Interactions that include a trajectory endpoint were excluded as we cannot guarantee these to be complete. Segment pairs s_1, s_2 were sorted by time. We assume the droplet creating s_1 to move freely, such that s_1 can be safely approximated by a straight line. We note that our swimmers' dynamics are persistent Brownian rather than strictly ballistic, however, the trajectory persistence length, as seen in Fig. S2 , clearly exceeds the typical interaction length, so that our assumption of straight segments is reasonable. We identified the orientation $\hat{\mathbf{e}}_1$ of s_1 via a linear regression fit. If the standard deviation of this fit exceeded a certain value, the segment was considered to be too crooked, i.e. not relating to free motion, and the interaction was discarded. Fig. S2 shows trajectories from one experimental run with the numerically identified interactions marked in color. We further discarded, by visual inspection, any interactions that were disturbed by multiple trail collisions. For segment s_2, we extracted the following quantities:

1. the time $\Delta t = t_2 - t_1$ elapsed between the two points of closest trajectory approach, which we chose as the time delay of the interaction.

2. for each coordinate in s_2, the distance to the closest point in s_1, i.e. distance $d(t)$ of swimmer to trail over time. To mark crossing events, by convention, d is signed via $\mathrm{sgn}(d) = \mathrm{sgn}(\mathbf{n}_2 \times \hat{\mathbf{e}}_1)$, with $\mathbf{n}_2$ denoting the s_2 trajectory normal and $\times$ the 2D cross product.

3. from a projection of $\mathbf{v}_2$ on $\hat{\mathbf{e}}_1$, droplet speeds $v_\parallel$ parallel and $v_\perp$ perpendicular to the trail, as well as the angle θ between $\mathbf{v}_2$ and $\hat{\mathbf{e}}_1$. To avoid discontinuities in θ due to 2π periodicities, θ was calculated with respect to $-\hat{\mathbf{e}}_1$ if $\langle v_\parallel \rangle > 0$.

We note that for non-specular reflections with $\alpha_0 \neq 0$ (Eqn. 1) the time of maximum rotation rate, $t_{\mathrm{turn}} = |\mathrm{d}\theta/\mathrm{d}t|_{\max}$, should be slightly delayed with respect to the time of closest approach.

F. Laser sheet fluorescent microscopy

The light sheet /selective plane fluorescent microscopy setup (Fig. S3) consists of an illumination unit producing the thin laser sheet and a detection unit, *i.e.* camera and objective. Both units are translated vertically by synchronized z-stages, capturing images at a frame size of 1MP in the xy plane at 4.15 μm/px resolution. Images were recorded at 150 frames per second, while the z-stages were driven by a sawtooth signal with an

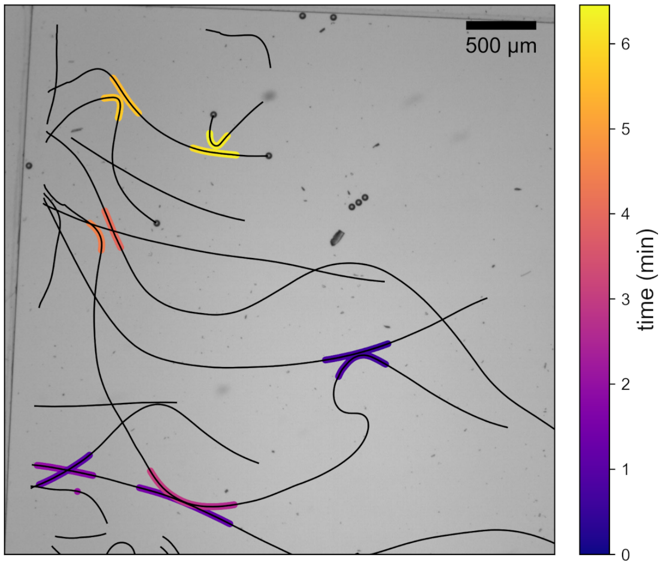

FIG. S2. Identification of collision events, experiment at low number densities. Black lines: extracted trajectories, detected collision events underlaid in time-encoded color. Entrained droplets are excluded from the analysis. Background: final frame in experiment.

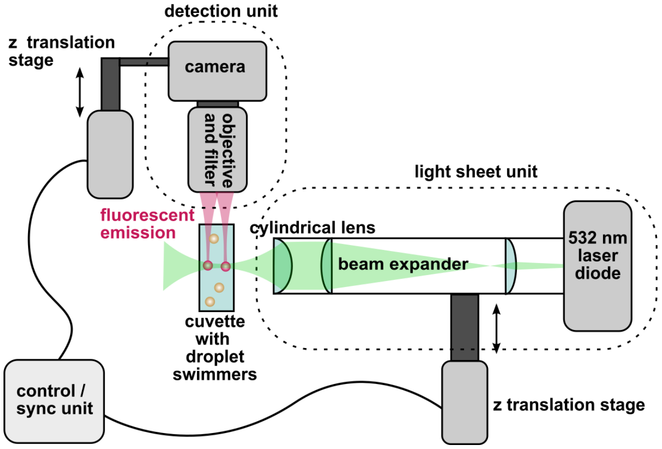

FIG. S3. Fluorescent light sheet microscope, schematic.

amplitude of 3 mm and a frequency of 0.7 Hz, yielding a voxel size of $4.2\text{x}4.2\text{x}27\,\mu m^3$. The laser sheet's beam waist is $\approx 40\,\mu m$ thick. Using a glass cuvette of internal size $3\text{x}3\,mm^2$, the accessible scanned sample volume is $3\text{x}3\text{x}3\,mm^3$. Our samples consisted of Nile Red doped droplets with a diameter of $50\,\mu m$ in density matched mixtures of TTAB/H_2O/D_2O.

3D droplet positions were reconstructed from binarized stacks of z-slices for each half period of the sawtooth signal. We extracted fluorescent droplet contours for each slice [2] and grouped contours associated with the same droplet within consecutive slices using a mean shift clustering algorithm [4, 5]. Time and z position for each slice were calculated using timestamps provided by the camera and translation stage software interfaces, resulting in

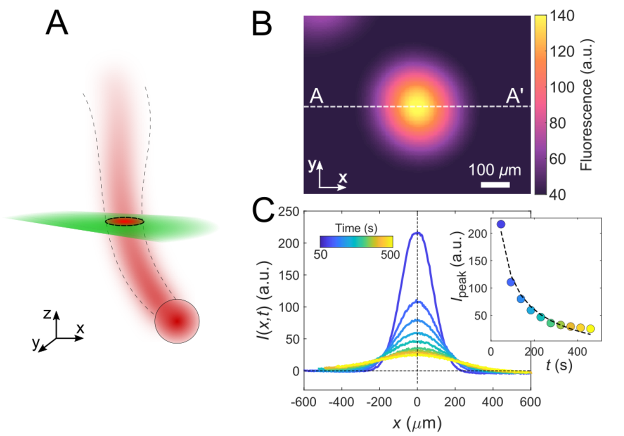

FIG. S4. Visualisation of the trail in 3D. **(a)** The schematic of trail visualisation in 3D. The green sheet represents the laser light which scans the trail at different heights obtaining images like **(b)**. **(c)** The temporal evolution of intensity profiles obtained by laser sheet measurements. The inset shows the peak values versus time, the dashed line is a fit to a $t^{-1/2}$ decay.

full $xyzt$ datasets.

We also validated the diffusive spreading of the trail in 3D: we first captured the time-dependent fluorescence in the trail of a droplet sedimenting under gravity along the light sheet normal, taken at a fixed sheet position in z (Fig. S4 a). We extracted the fluorescence intensity profile $I(x,t)$ of a cross section of the trail along the line AA′ (Fig. S4 c), as shown in the false-color sample image in Fig. S4 b. The peak values I_{peak} scale with $t^{-1/2}$ (inset), similar to the 2D behavior analyzed in Fig. 1.

G. Modeling of individual droplet-trail interactions

We model the micelle trail left by a preceding droplet as a chemical field c with Gaussian profile perpendicular to the direction of motion, with a width that depends on the time Δt elapsed since the preceding droplet moved away from the point of interest. Without loss of generality we assume the preceding droplet to propel along x. Because the diffusion of the micelles is slow compared to the propulsion velocity of the droplets ($D_{\text{fm}}/(\Delta t V_0^2) \ll 1$), we can neglect the x-dependence of the concentration profile, as well as its time-dependence, so that the chemical field is given by the static, one-dimensional profile

$$c(y) = \frac{c_0}{\sqrt{4\pi D_{\text{fm}}\Delta t}} \exp\left(-\frac{y^2}{4D_{\text{fm}}\Delta t}\right), \qquad \text{(S5)}$$

where D_{fm} is the diffusion coefficient of the (filled) micelles. Here, c_0 is a constant with units of inverse area, which can be related to the system parameters as $c_0 = \lambda/(V_0 h)$, where λ is the rate of micelle release by the droplets, V_0 the droplet propulsion velocity, and h the height of the Hele-Shaw cell.

We combine (S5) with the CAPP model in Eqs. (1) and (2) of the main text, and further ignore translational and rotational diffusion as they are both negligible within the short time scale of a single interaction event. We thus solve the following deterministic equations for the incoming droplet's position (x, y) and orientation θ, which is defined as the angle between $\boldsymbol{n}$ and the x-axis (see Fig. 2D):

$$\dot{x} = V_0 \cos\theta \qquad \text{(S6)}$$

$$\dot{y} = V_0 \sin\theta + \frac{\alpha c_0 y}{\sqrt{2\pi}(2D_{\text{fm}}\Delta t)^{3/2}} \exp\left(-\frac{y^2}{4D_{\text{fm}}\Delta t}\right) \qquad \text{(S7)}$$

$$\dot{\theta} = -\frac{\Omega c_0 y}{\sqrt{2\pi}(2D_{\text{fm}}\Delta t)^{3/2}} \exp\left(-\frac{y^2}{4D_{\text{fm}}\Delta t}\right) \cos\theta. \qquad \text{(S8)}$$

H. Fitting of individual droplet-trail interactions

For the diffusion coefficient of the micelles, we used the measured value $D_{\text{fm}} = 52.5\,\mu\text{m}^2\,\text{s}^{-1}$. The droplet velocity V_0 was estimated by the average velocity in the given trajectory, and the experimentally-measured time lag Δt was used as an input. Initial conditions for the time evolution of $(x(t), y(t), \theta(t))$ were obtained from the data as described in the previous section: $y(0) \approx 200\,\mu\text{m}$ corresponds to the initial value of the signed distance d_{max}, $\theta(0)$ to the incidence angle, and $x(0) = 0$ without loss of generality. Thus, the only unknown parameters are the two products Ωc_0 and αc_0, which are used as fit parameters.

For our analysis, we focused on sharp reflection events with $\theta_{\text{inc}} > 60°$, for which our minimal model of the trail as a static Gaussian with x-independent width is best justified (for low incidence angles, interactions occur over longer times and a wider range of x-values, which may affect the validity of our approximation and introduce differences between parallel and antiparallel reflection events). The 54 reflection events were fitted and ordered from best to worst fit according to the fit error. By analyzing the median values of Ωc_0 and αc_0 calculated from the n best fits as a function of n, we found that the median values stabilize at $\Omega c_0 \approx 7 \times 10^3\,\mu\text{m}^2\,\text{s}^{-1}$ and $\alpha c_0 \approx 3 \times 10^4\,\mu\text{m}^3/\text{s}$ between $n \approx 20$ and $n \approx 40$. Smaller n values are susceptible to the noise due to small number statistics, whereas for larger n we would include bad fits that skew the distribution, presumably corresponding to non-ideal trajectories (e.g. interactions with curved trails or measurement artefacts due to global drift in the chamber). Details can be found in Fig. S5. We note that we do not distinguish between parallel and antiparallel interactions: for $\theta_{\text{inc}} > 90°$, we convert to $180° - \theta_{\text{inc}}$.

To obtain an approximate transition boundary (the experimental separatrix in Fig. 3) between the experimen-

tal reflecting and crossing events we binned the Δt and θ_{inc} data sets to ten and nine groups, respectively. Each reflecting data point was assigned a value of 1 and each crossing data point was assigned a value of 0. Then, we calculated the average value in each bin. We fit a spline curve to the data sets in each Δt bin to obtain the transition point which was defined as the θ_{inc} at which the curve reaches 0.5. We then fit a sigmoid function to the 8 data points (the black dashed line in Fig. 3). The fitting resulted in a root mean square error of RMSE = 10.5% which we used to plot the error band covering the region where the reflecting and crossing events coexist. For $\Delta t < 50$ s which is broader than the relevant range for the caging experiments ($\Delta t < 20$ s), there is a good agreement between between the experimental and the theoretical separatrices.

I. Modeling of collective behavior

To model the behavior of a many-droplet system with N droplets, we use the equations of the CAPP model, Eqs. (1) and (2) of the main text with $i = 1, ..., N$, together with an evolution equation for the concentration field $c(\boldsymbol{r}, t)$ of the filled micelles

$$\partial_t c(\boldsymbol{r}, t) = D_{\mathrm{fm}} \nabla^2 c + \lambda \sum_{j=1}^{N} \delta(\boldsymbol{r} - \boldsymbol{r}_j) \tag{S9}$$

where λ is the rate of micelle production by the droplets, which are modeled as point sources. Eq. (S9) can be solved by considering a superposition of instant point sources released at all times $t' < t$, at locations $\boldsymbol{r}_j(t')$ for all $j = 1, ..., N$, each of which undergoes two-dimensional diffusion:

$$c(\boldsymbol{r}, t) = \frac{\lambda}{h} \sum_{j=1}^{N} \int_{-\infty}^{t} \mathrm{d}t' \frac{\exp\left(-\frac{(\boldsymbol{r}-\boldsymbol{r}_j(t'))^2}{4D_{\mathrm{fm}}(t-t')}\right)}{4\pi D_{\mathrm{fm}}(t-t')} \tag{S10}$$

Its gradient can be directly calculated as

$$\nabla c(\boldsymbol{r}, t) = -2\frac{\lambda}{h} \sum_{j=1}^{N} \int_{-\infty}^{t} \mathrm{d}t' \frac{[\boldsymbol{r} - \boldsymbol{r}_j(t')] \exp\left(-\frac{(\boldsymbol{r}-\boldsymbol{r}_j(t'))^2}{4D_{\mathrm{fm}}(t-t')}\right)}{\pi[4D_{\mathrm{fm}}(t-t')]^2} \tag{S11}$$

The combination of Eqs. (1) and (2) in the main text with (S11) gives a closed set of evolution equations for the system. However, as a consequence of assuming the particles to be point sources and to respond to the local gradient, this model leads to spurious, infinitely-strong self-interactions of each droplet with the micelles it just released. These can in principle be regularized by considering the finite size of droplets. Nevertheless, because we are interested in describing collective behavior in systems with a large number of particles, we can side-step this problem by simply neglecting the self-interaction and defining the local gradient that enters Eqs. (1) and (2) as

$$\nabla c|_{\boldsymbol{r}=\boldsymbol{r}_i} = -2\frac{\lambda}{h} \sum_{\substack{j=1 \\ j\neq i}}^{N} \int_{-\infty}^{t} \mathrm{d}t' \frac{[\boldsymbol{r}_i - \boldsymbol{r}_j(t')] \exp\left(-\frac{(\boldsymbol{r}_i-\boldsymbol{r}_j(t'))^2}{4D_{\mathrm{fm}}(t-t')}\right)}{\pi[4D_{\mathrm{fm}}(t-t')]^2} \tag{S12}$$

Using (S12), we numerically solve equations (1) and (2) using Brownian dynamics using the parameters V_0, D_{fm}, D_t and D_r as measured experimentally, and the parameters Ωc_0 and αc_0 as extracted from the fits to individual interaction events. The simulations use an Euler time step of 10^{-2} s, and a square box with periodic boundary conditions with minimum-image convention for all distances $\boldsymbol{r}_i - \boldsymbol{r}_j(t')$ in (S12). The results in Fig. 4D of the main text used a box of side length $L = 6.32$ mm and particle numbers $N = 1, 72, 148, 296, 343$, resulting in the same particle area densities as in the experiments, and the trajectory in Fig. 4D corresponds to the simulation with $L = 6.32$ mm and $N = 343$.

In the experimental data, since ballistic runs are uncorrelated, the long-time dynamics presumably correspond to a diffusive random walk, however, it is not feasible to reliably quantify the respective exponent in the experimental MSD due to the limited lifetime of the droplets and the finite size of the experimental cell.

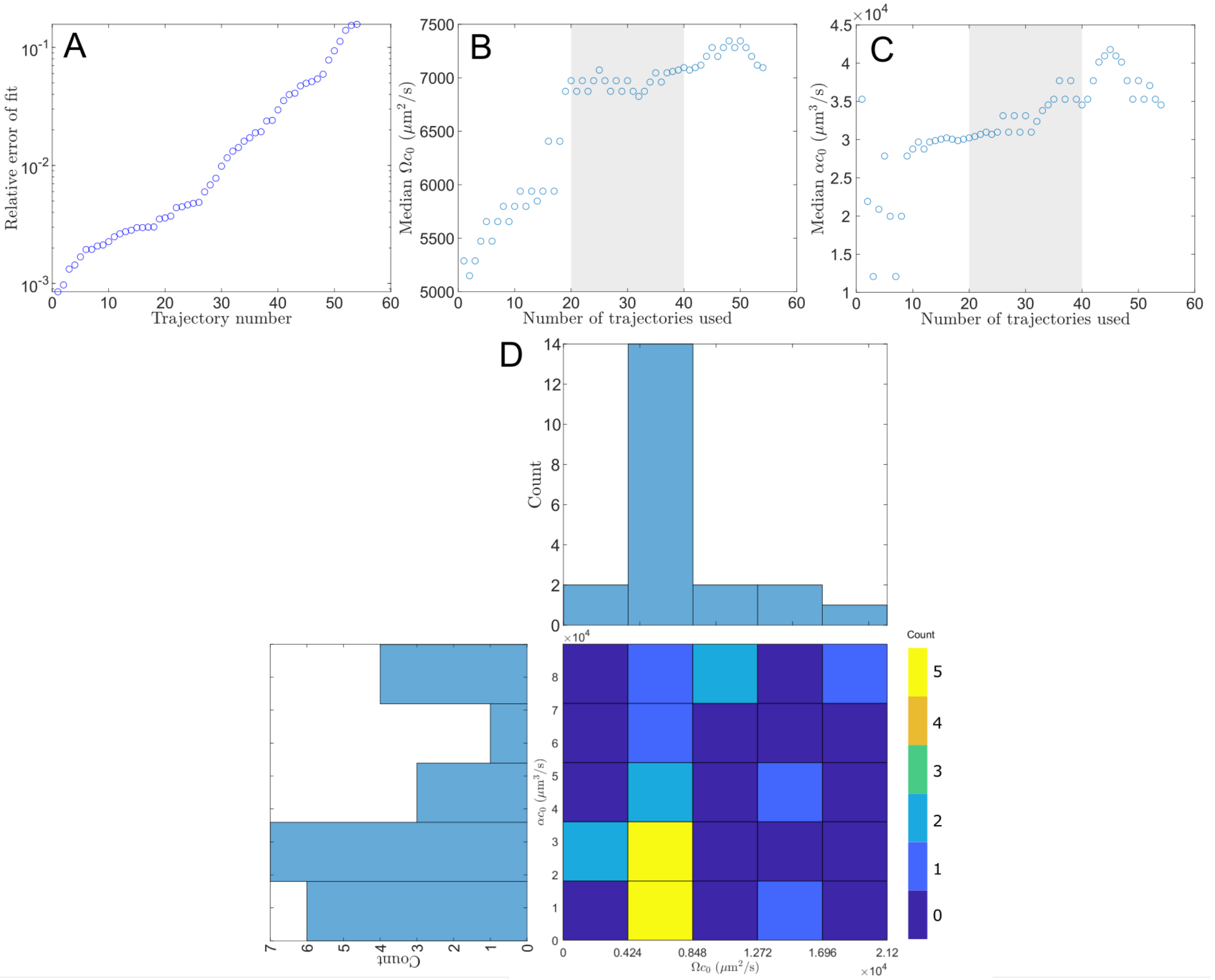

FIG. S5. Analysis of the fits to the 54 experimental reflection events with large incidence angle $\theta_{\rm inc} > 60^\circ$, using two fitting parameters Ωc_0 and αc_0. (a) The fits are ordered from best to worst according to the error of the fit. (b,c) Median values of (b) Ωc_0 and (c) αc_0 as a function of the the number n of trajectories used in the calculation of the median value. We observe a plateau at $\Omega c_0 \approx 7 \cdot 10^3$ μm^2/s and $\alpha c_0 \approx 3 \cdot 10^4$ μm^3/s (gray band), when a sufficient number of trajectories is considered ($n \gtrsim 20$) but the worst fits are left out ($n \lesssim 40$). (d) As an example, we show the histogram for the values of Ωc_0 and αc_0 (two-dimensional, as well as projected along each dimension) when using the $n = 21$ best fits, which corresponds to using only the fits with relative error $< 4 \cdot 10^{-3}$.

2. SUPPLEMENTAL VIDEOS

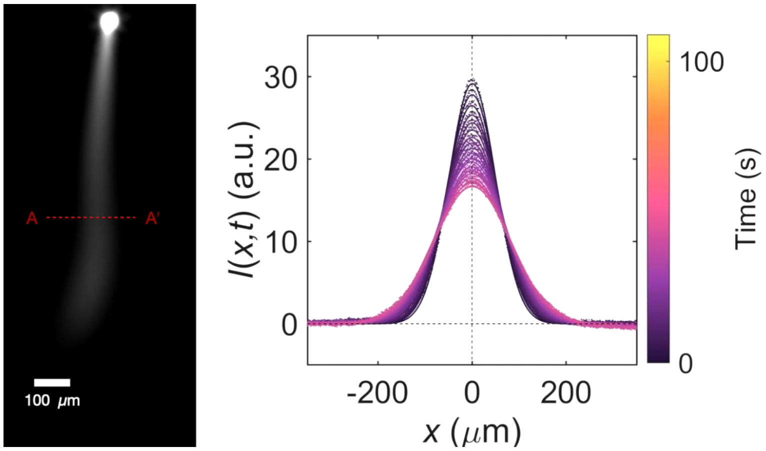

Supplemental Video S1: Quantitative imaging of the chemical trail of a droplet via fluorescence microscopy. The animated plot visualizes the fluorescence measured at the same time along the line AA' in the video on the left. Video sped up 10x. The swimming medium is 5 wt.% TTAB solution.

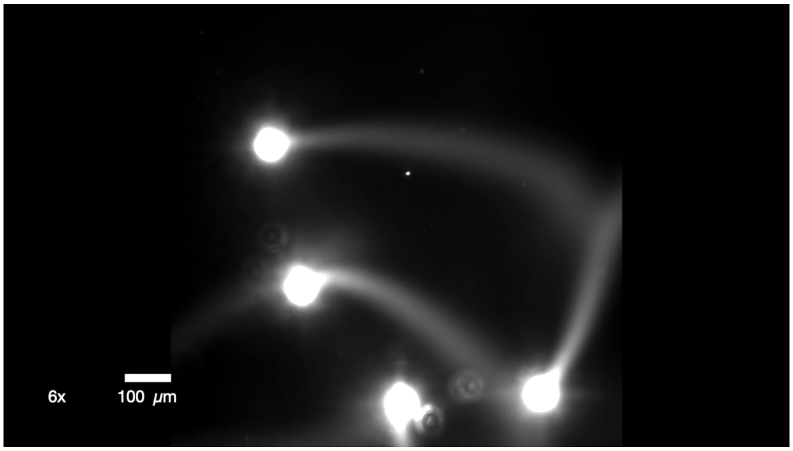

Supplemental Video S2: Autochemotactic interaction between a droplet and a trail: Crossing events. Fluorescent videomicroscopy of NileRed dyed droplet swimmers, with the dye comigrating into the swimmer trails. The swimming medium is 5 wt.% TTAB solution.

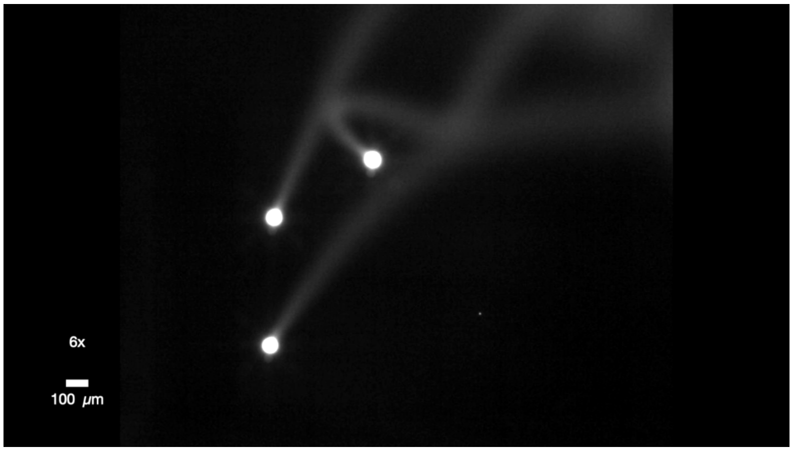

Supplemental Video S3: Autochemotactic interaction between a droplet and a trail: Reflecting events. The illumination is changed back and forth between the fluorescence and visible light. The swimming medium is 5 wt.% TTAB solution.

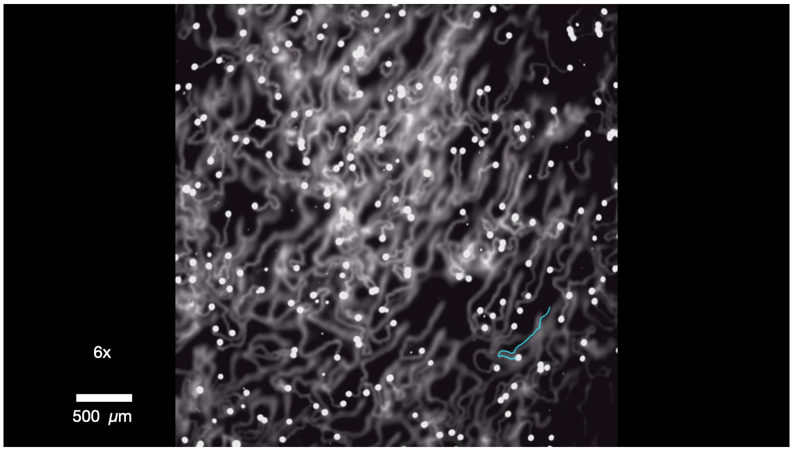

Supplemental Video S4: 2D autochemotactic caging in a Hele-Shaw (quasi-2D) cell, observed under fluorescent microscopy. The TTAB concentration was increased to 25 wt.% compared to the experiments used for quantitative analysis (5 wt.%), for better trail visibility. The less persistent swimming is due to the higher solubilization rate. Stills from this video were used in Fig. 3.

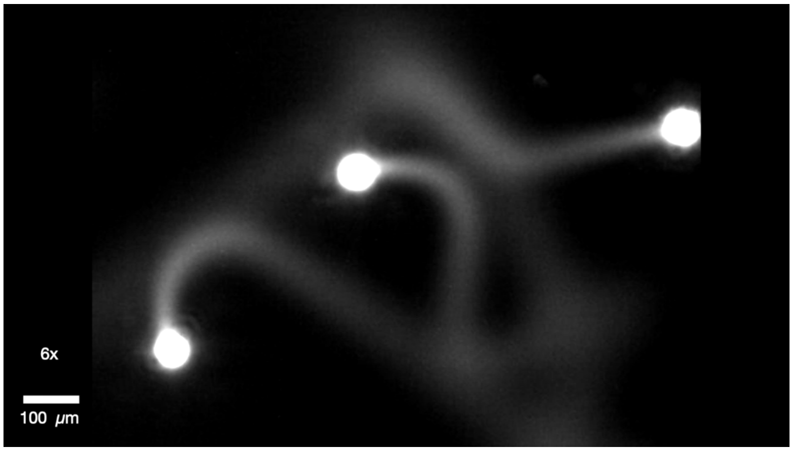

Supplemental Video S5: An example caging event in a *dilute* active emulsion followed by a "cage escape", observed under fluorescence microscopy. The swimming medium is 5 wt.% TTAB solution. The red arrows point to the position of the reflection events.

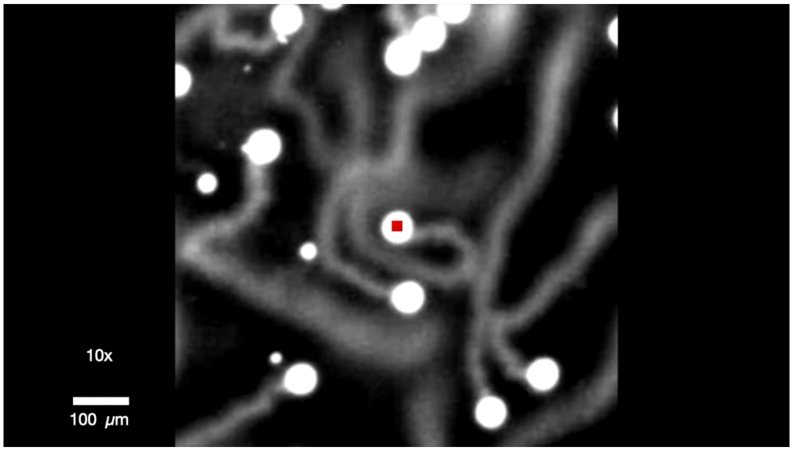

Supplemental Video S6: Cage escape due to the chemical buildup within a *dense* active emulsion, observed under fluorescence microscopy. The droplet, marked red, is tracked and centered in the movie frames. The swimming

medium is 25 wt.% TTAB solution.

[1] B. V. Hokmabad, R. Dey, M. Jalaal, D. Mohanty, M. Almukambetova, K. A. Baldwin, D. Lohse, and C. C. Maass, Physical Review X **11**, 011043 (2021).
[2] G. Bradski, Dr Dobbs J. Softw. Tools **25**, 120 (2000).
[3] J. C. Crocker and D. G. Grier, Journal of Colloid and Interface Science **179**, 298 (1996).
[4] F. Pedregosa, G. Varoquaux, A. Gramfort, V. Michel, B. Thirion, O. Grisel, M. Blondel, P. Prettenhofer, R. Weiss, V. Dubourg, J. Vanderplas, A. Passos, D. Cournapeau, M. Brucher, M. Perrot, and É. Duchesnay, Journal of Machine Learning Research **12**, 2825 (2011).
[5] D. Comaniciu and P. Meer, IEEE Transactions on Pattern Analysis and Machine Intelligence **24**, 603 (2002).
